# Coral reefs in the Mahafaly Seascape (SW Madagascar) as potential climate refugia following the 2024 mass bleaching event

**DOI:** 10.7717/peerj.20319

**Published:** 2025-11-25

**Authors:** Mahery Randrianarivo, Solotahiana Rakotomanga, Fanja Razafindramasy, Danny Kornelio Ravelojaona, Tahiry Ranaivoson, Domoina Rakotomalala, Rémi Ratsimbazafy

**Affiliations:** World Wide Fund For Nature, Madagascar Country Office (WWF MDCO), Antsakaviro, Antananarivo, Madagascar

**Keywords:** Climate change, Thermal stress, Coral bleaching, Recovery potential, Locally managed marine areas, Southwestern Indian Ocean

## Abstract

The southwesternmost coast of Madagascar, including the Mahafaly seascape, is home to some of the country’s most ecologically and culturally important coral reefs, which remain largely understudied. These ecosystems are facing growing pressure from a panel of disturbances, with climate-induced events such as marine heatwaves being among of the causes of coral bleaching. The decline of these reefs can directly threaten the livelihoods of the local communities, who depend heavily on the resources they provide. In this context, understanding how different disturbances impact coral reef condition and recovery is critical for developing effective management strategies and conservation for this vulnerable region. This study investigates coral bleaching dynamics in the Mahafaly seascape during and after the fourth mass coral bleaching event in 2024. Six reef locations within locally managed marine areas were surveyed, comprising a total of 11 stations, with 20 one-square-meter quadrats randomly deployed at each station during each sampling period. Average hard coral densities of 18.3 and 19.3 colonies m^−2^ were recorded during and after the event, respectively. Bleaching prevalence peaked during the event, affecting 38.8% of coral colonies, with a marked decrease to 6.0% afterward. However, coral mortality remained relatively low across the entire seascape, with a slight post-bleaching increase in dead colonies (+3.5%). Northern sites experienced the highest thermal stress, with Beheloke and Besambay reefs being the most susceptible to bleaching, whereas Ambola exhibited remarkable resilience. In the south, Ambohibola showed low vulnerability, Lembehitake moderate susceptibility, and Itampolo the highest bleaching prevalence despite lower thermal stress. Taxa-specific patterns were also evident, with thermally sensitive branching genera being most affected, contrasting with the relative tolerance of massive and encrusting corals. The heterogeneous coral bleaching patterns observed across the seascape emphasize the importance of site- and taxa-specific monitoring to prioritize management actions where reef resilience is highest or degradation most severe. Despite bleaching severity, the results suggest that coral assemblages in the seascape have a strong capacity to recover following the event. The Mahafaly seascape reefs could serve as vital refugia in the face of climate change, underscoring the need for ongoing conservation efforts.

## Introduction

Coral reefs are among the most biologically diverse and dynamic ecosystems, essential for the well-being of nearly 850 million people in over 100 countries, particularly in developing nations (Woodhead et al., 2019). Healthy coral reef ecosystems rely on scleractinian corals, which serve as the structural foundation and key engineers of these systems (Vuleta, Nakagawa & Ainsworth, 2024). Due to their dominant sessile life cycle and high sensitivity, corals are particularly exposed to environmental changes, especially compared to more mobile and resilient organisms. Despite enduring climatic oscillations and mass extinctions through ecological time, coral reefs have maintained their functional role (Kinzie III & Buddemeier, 1996). However, their historical resilience is increasingly challenged by the growing frequency and intensity of both natural and anthropogenic disturbances in the Anthropocene (Andrello et al., 2022). The impact of global climate change on these ecosystems is most apparent through bleaching, a physiological response to environmental stress such as elevated sea surface temperatures, which disrupts the symbiosis between the Cnidarian host (also occurring in other taxa, *e.g.* some Foraminifera, Porifera and Mollusca) and their endosymbiotic algae from the Symbiodinaceae family (LaJeunesse et al., 2018). This latter is responsible for coral tissue pigmentation and supplies up to 95% of the coral’s energy needs (Muscatine, 1990). While bleaching is a reversible phenomenon and corals have the potential to recover, prolonged or repeated disruptions in the coral-algae relationship can lead to the eventual death of the bleached corals (Voolstra et al., 2024). Global change, which amplifies the differences between each El Niño and Southern Oscillation (ENSO) phase, is expected to cause extreme events to double in frequency in the future (Cai et al., 2020). Indeed, in just two decades, we have already observed an earlier onset, greater severity, and temporal persistence of this event (Cai et al., 2023).

The fourth global coral bleaching event was confirmed in early 2024, the current warmest year on record, with unprecedented coral mortality predicted in the Indo-Pacific (Hoegh-Guldberg et al., 2023; Reimer et al., 2024). When the first global event occurred in 1998, 16% of corals perished (Wilkinson, 2008). The second event, in 2010, impacted reefs in over 60 countries (Donner, Rickbeil & Heron, 2017), while the third, which persisted from 2014 to 2017, affected over 70% of the global coral reefs (Hughes et al., 2018a). Overall, hard coral cover at global scale declined from 36% to 19% between the first and the third mass bleaching event (Tebbett, Connolly & Bellwood, 2023). As a result of these recurring events, many coral reefs have undergone a striking phase shift, where dominant reef-building corals are replaced by macroalgae or other non-reef-building benthic organisms, such as cyanobacteria. These shifts vary across regions (Roff & Mumby, 2012), but generally represent undesirable states that provide fewer ecosystem goods and services to human populations (Eddy et al., 2021).

Coral bleaching is evolving into a highly dynamic phenomenon, both spatially and temporally (Mellin et al., 2024). Furthermore, susceptibility to bleaching differs among coral species and their endosymbionts. Generally, stress-tolerant corals exhibit greater resistance, whereas competitive and weedy species, characterized respectively by fast-growing or opportunistic traits, are usually more vulnerable (Darling, McClanahan & Côté, 2013). Within taxa, larger colonies and corals with branching or tabular growth form often exhibit higher bleaching severity and mortality (Penin et al., 2007; Obura, Bigot & Benzoni, 2018; Winslow et al., 2024). The impacts of bleaching are typically lower at greater depths and in areas where other factors help offset or reduce sea surface temperature (SST) warming (Penin et al., 2007). However, some studies indicate that this are not always the case, as bleaching response on many reefs changes over time due to a combination of differential survival of genotypes, changing species dominance, acclimation to thermal stress, and characteristics of local induced stressors (McClanahan et al., 2007a; Grottoli et al., 2014; Guest et al., 2016; McClanahan, 2022; Hughes et al., 2018b; Szereday, Voolstra & Amri, 2024). This increasing variability complicates efforts to predict when and where bleaching will occur, as well as how reefs will respond. Consequently, the development of uniform management strategies becomes difficult, requiring more adaptive approaches that account for these dynamic patterns.

Madagascar’s coastline is surrounded by approximately 2,400 km^2^ of coral reefs, recognized as a hotspot for marine biodiversity in the southwest Indian Ocean (Cooke, 2012). Over 6,000 marine organisms have been recorded in the island, including 380 species of scleractinian corals (Veron & Turak, 2003). Madagascar’s reefs are essential for the estimated 13 million people who live along its coasts, providing crucial economic, cultural, and social ecosystem services primarily through fisheries (Behivoke et al., 2021). The high dependence on reef resources, along with environmental destruction upstream, has contributed to the decline of these reefs over the past 50 years (Bruggemann et al., 2012; Maina et al., 2013). As with most reefs worldwide, those in Madagascar have suffered from climate change, with widespread bleaching reported during both the first and third global events, causing more severe impacts on west coast reefs compared to the east (McClanahan & Obura, 1998; Bigot et al., 2000; Ahamada et al., 2002; McClanahan et al., 2007b; McClanahan et al., 2019; Gudka et al., 2020).

Despite documented coral bleaching events in Madagascar, previous studies have relied on single surveys, often overlooking spatial variations across different reef environments and the subsequent impacts. To address this gap, we report on the bleaching pattern of coral assemblages across several reef sites during and immediately after the fourth mass coral bleaching event in 2024. Our study focuses on the Mahafaly seascape in southwest Madagascar, where Locally Managed Marine Areas (LMMAs) play an important role in managing fishing activities in the coral reef area, and ecologically, for reinforcing buffer and peripheral zones of existing Marine Protected Areas (Ratsimbazafy et al., 2019). This study aims to (*a*) assess the spatiotemporal variation in the response of coral assemblages to bleaching within the Mahafaly seascape, and (*b*) identify patterns of recovery and mortality to inform future conservation strategies and management practices within LMMAs.

## Materials & Methods

### Study area

**Figure 1:**
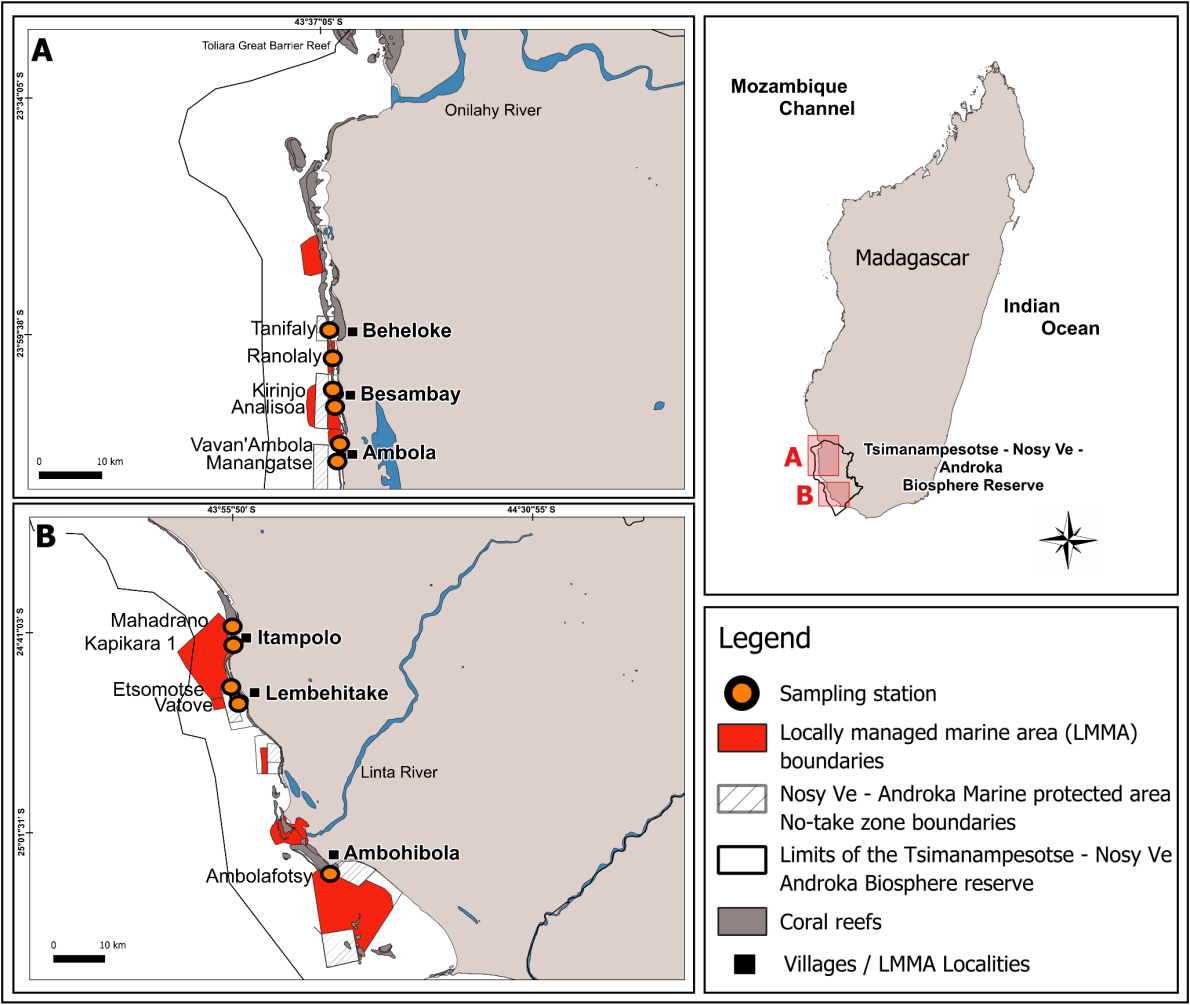
Map of the Mahafaly Seascape, within the Tsimanampesotse–Nosy Ve Androka Biosphere Reserve. The map shows the boundaries and locations of the six surveyed Locally Managed Marine Areas (LMMAs), the 11 sampling stations, and the no-take zone boundaries of the Nosy Ve–Androka Marine Protected Area (MPA).

This study was conducted in the Mahafaly seascape reefs, located between the Onilahy River (23°34′S) and the Linta River (25°01′S), part of a larger reef system in the southwest of Madagascar (Fig. 1). Coral reefs in this seascape span ∼28,000 hectares and host 140 species of scleractinian corals, with an average hard coral cover of 32% as of early 2024 (WWF MDCO, 2024, unpublished data).

This area is included within the marine section of the Tsimanampesotse-Nosy Ve Androka Biosphere Reserve, designated in 2018. This Biosphere Reserve is part of the intertropical marine biogeographic system of the Southwest Indian Ocean (SWIO), encompassing five major Malagasy ecoregions: the South Terrestrial Ecoregion, the Aquatic Ecoregion of the Southern Basins, the Aquatic Ecoregion of the West Basins, the Mozambique Channel Marine Ecoregion, and the South Marine Ecoregion (UNESCO, 2023). The Biosphere Reserve includes the Nosy Ve-Androka Marine Protected Area (MPA), classified as a Category II area under IUCN guidelines, as well as 17 LMMAs, which help maintain the ecological integrity of its MPA.

The local population consists of two main ethnic groups: the *Vezo*, who live near the coast and primarily practice fishing, and the *Tanalana*, further inland and who focus on agropastoralism, as well as fishing during dry periods. Fishing on the reefs in southwest Madagascar has increased due to coastal population growth and rising market demand for seafood, with over 75% of the 34,300 coastal residents relying on small-scale fisheries as their main income source (MPRH, 2012).

### Benthic surveys

Our sampling design was informed by the 2016 mass coral bleaching event, when peak coral bleaching in the SWIO reefs occurred in March, decreased by May, and subsided by the austral winter (Gudka et al., 2020). To capture this temporal variation, we surveyed the bleaching of scleractinian corals and the calcareous hydrocoral *Millepora*, during two periods: March and August 2024, hereafter mentioned as “during” and “after” the event. Surveys were conducted at 11 stations, across six reef sites within LMMA: Beheloke, Besambay, Ambola, Itampolo, Lembehitake, and Ambohibola (from north to south; Fig. 1, Table 1). Two sampling stations were established at each site. These two stations were placed on the outer slope at a depth of ∼10 m. The exception is for Ambohibola, which had only one station located on an inner reef at a depth of ∼5 m.

**Table 1:** Characteristics of the eleven sampling stations and six reef sites within Locally Managed Marine Areas (LMMAs).

Site within LMMA	LMMA creation date	LMMA area (Ha)	Number of implied villages, population and fishers	Sampling station	Lat.	Long.
Beheloke	2008	533	7 villages, 1,221 populations	Ranolaly	23°56′27″S	43°39′9″E
				Tanifaly	23°53′49″S	43°38′42″E
Besambay	2012	1,774	3 villages; 548 populations; 324 fishers	Analisoa	24°00′04.38″S	43°39′.32.24″ E
				Kirinjo	23°59′21″S	43°39′46″E
Ambola	2012	12	8 villages; 827 populations; 167 fishers	Vavan’Ambola	24°4′26″S	43°40′12″E
				Manangatse	24°4′45″S	43°39′58″E
Itampolo	2007	10,917	6 villages; 5, 609 populations	Kapikara 1	24°41′35″	43°55′49″
				Mahadrano	24°39′34″	43°55′48″
Lembehitake	2008	483	11 villages, 267 populations, 205 fishers	Etsomotse	24°46′48″S	43°55′44″E
				Vatove	24°47′14″S	43°56′38″E
Ambohibola	2007	15,649	6 villages, 890 populations, 113 fishers	Ambolafotsy	25°5′44″S	44°7′10″E

**Figure 2:**
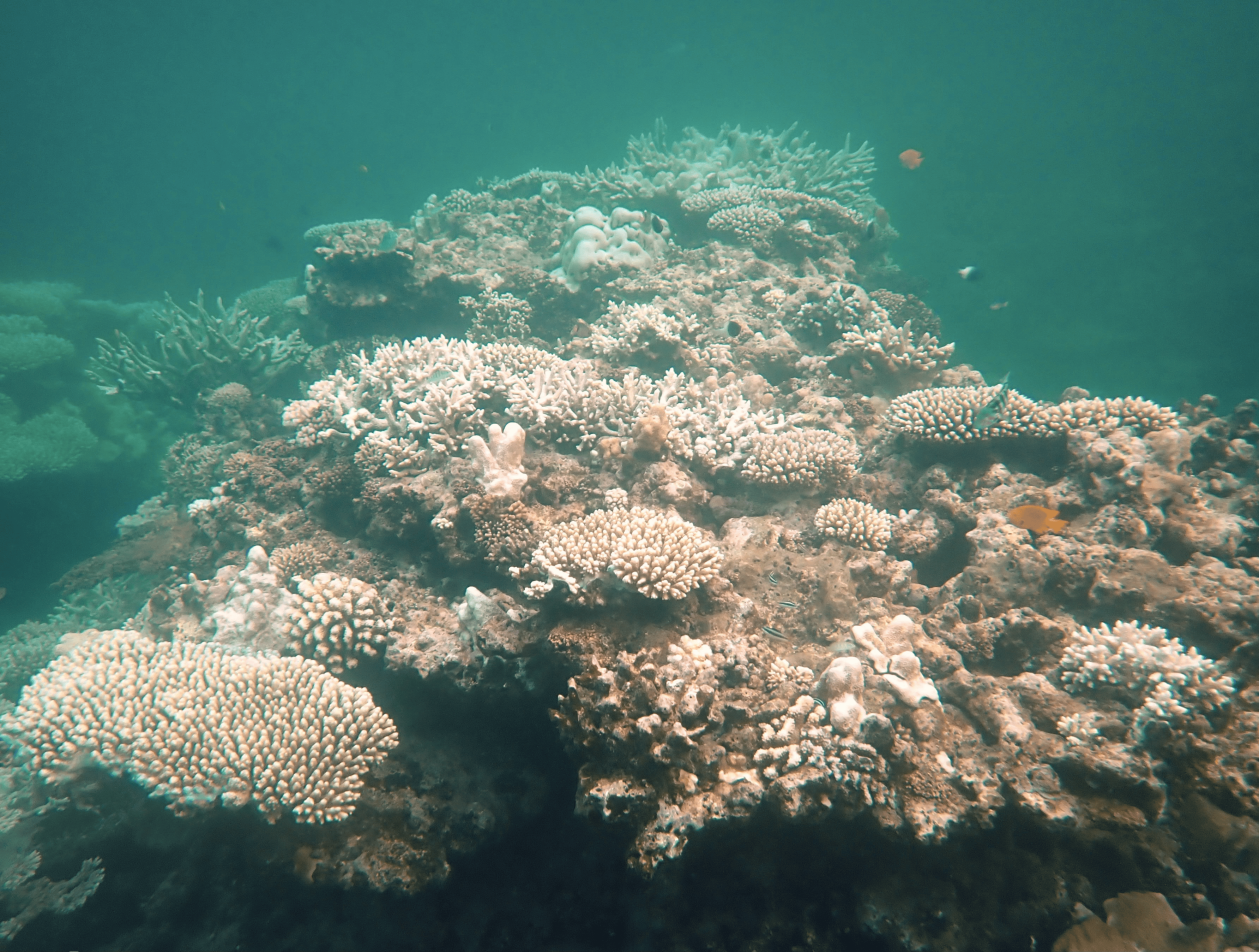
Photograph of coral colonies, most exhibiting extreme bleaching level (C5: >90%) during the event at Itampolo (Mahadrano Station).

At each station, 20 quadrats of 1 m^2^ (1 m × 1 m) were haphazardly deployed. All coral colonies within each quadrat were identified to the genus level, and their bleaching status was recorded using the following categories, as adapted by Gudka et al. (2020) from McClanahan (2004): *Healthy* (C1: less than 1% of the colony bleached or dead), *Low* (C2: 1–10%), *Medium* (C3: 10–50%), *High* (C4: 50–90%), *Extreme* (C5: more than 90%, *e.g.* Fig. 2), and *Dead* (C6: combination of recently dead, identified as those covered by turf algae or microbial growth, and long-dead corals, consisting only of skeletons).

The severity and susceptibility to bleaching for coral taxa and reef site were assessed using the methodology described in McClanahan (2004). For each coral genus, we calculated the percentage of colonies falling into the six defined bleaching categories. Numerical weights were assigned to each category, ranging from 0 (C1) to 5 (C6), reflecting increasing levels of bleaching severity. The Bleaching Response Index (BRI) was then calculated using a weighted average according to Eq. (1):

(1) \begin{eqnarray*}BRI= \frac{0C1+1C2+2C3+3C4+4C5+5C6}{5} .\end{eqnarray*}

This division by 5 normalizes the index on a scale from 0 to 100, where higher values indicate greater bleaching severity for a given taxon or site. The BRI for a site was calculated by summing all colonies BRI of all genera during each sampling period and applying Eq. (1).

The site-specific Bleaching Susceptibility Index (BSI; McClanahan et al., 2007b) was determined by multiplying the taxa-specific BRI by its relative abundance, summing for all genera at the concerned reef site, and dividing by the total number of genera, according to Eq. (2):

(2) \begin{eqnarray*}\mathrm{BSI}=\sum _{i=1}^{N} \frac{BR{I}_{i}x{D}_{i}}{N} .\end{eqnarray*}

Here, *i* represents each coral genus, *D* is the relative abundance, and *N* is the total number of genera. Taxa with fewer than five observations were excluded, as minority taxa can significantly bias the BSI (Szereday, Voolstra & Amri, 2024).

Overall, the BRI reflects the intensity of bleaching for specific coral genera, while BSI offers an integrated measure of susceptibility across the entire coral community within a given reef site for each sampling period.

### Sea surface temperature (SST) and degree heating week (DHW)

Daily maximum SST and DHW for 2024 at 11 stations across the 6 reef sites were obtained from the NOAA Physical Sciences Laboratory (Reynolds et al., 2007; https://psl.noaa.gov/), NOAA Optimum Interpolation Sea Surface Temperature (OISST) V2 High-Resolution Dataset. DHW values were derived from HotSpot anomalies to estimate bleaching risk. They represent the cumulative temperature anomalies above the local maximum monthly mean (MMM) SST over a continuous 12-week period (Liu et al., 2017). A DHW of 4 ^∘^C-weeks was considered the threshold for widespread bleaching, corresponding to temperatures ≥ 1 ^∘^C above the long-term MMM sustained for four weeks (Whitaker & DeCarlo, 2024).

### Statistical analysis

We investigated the spatiotemporal variation in the abundance of coral colonies (all taxa pooled), considering their health status (three factors: Healthy, Bleached (representing all levels of bleaching), and Dead), across reef sites (six factors: Beheloke, Besambay, Ambola, Itampolo, Lembehitake, and Ambohibola), sampling periods (two factors: During and After bleaching event), and thermal stress predictors. We tested these effects and their relevant interactions using generalized linear mixed models (GLMMs) implemented in the *glmmTMB* package in R (Brooks et al., 2017), with Station included as a random effect. Multicollinearity among fixed effects was assessed using Variance Inflation Factors (VIFs) with the *performance* package in R (Lüdecke et al., 2021). The variable DHW was dropped from the GLMM due to rank deficiency in the conditional model. Apart from this, all VIFs were <2, indicating low correlation among the remaining predictors (Table S1). Nevertheless, thermal stress patterns were presented descriptively to support the interpretation of bleaching events.

The most parsimonious model was identified with the *MuMIn* R package (Bartoń, 2025). Based on the corrected Akaike Information Criterion, the best-supported model included site, sampling period, health status, and their two- and three-way interactions. Coral abundance was modelled using a negative binomial distribution, and significance of fixed effects in GLMMs was evaluated using likelihood ratio *χ*^2^ tests. Model fit and assumptions were assessed with simulated residuals using the *DHARMa* R package (Hartig, 2024), confirming adequate fit and absence of overdispersion or influential outliers (Fig. S1).

We then examined differences between factorial modalities involving more than two factors using Tukey’s post-hoc tests with *emmeans* R package (Lenth, 2025).

Particular attention was paid to *Acropora*, *Galaxea*, *Pocillopora*, *Stylophora*, *Echinopora*, *Favites*, *Seriatopora*, massive *Porites* and *Montipora* genera, which together represent over 80% of the total coral abundance in our dataset. The remaining, less representative genera were categorized as “Others”. To further explore patterns of recovery or mortality within these groups, we compared the relative abundance of bleached and dead corals between the two sampling periods using Student’s *t*-test analysis. All statistical analyses were performed using R 4.5.1 (R Core Team, 2025).

## Results

### Trends in thermal stress

**Figure 3:**
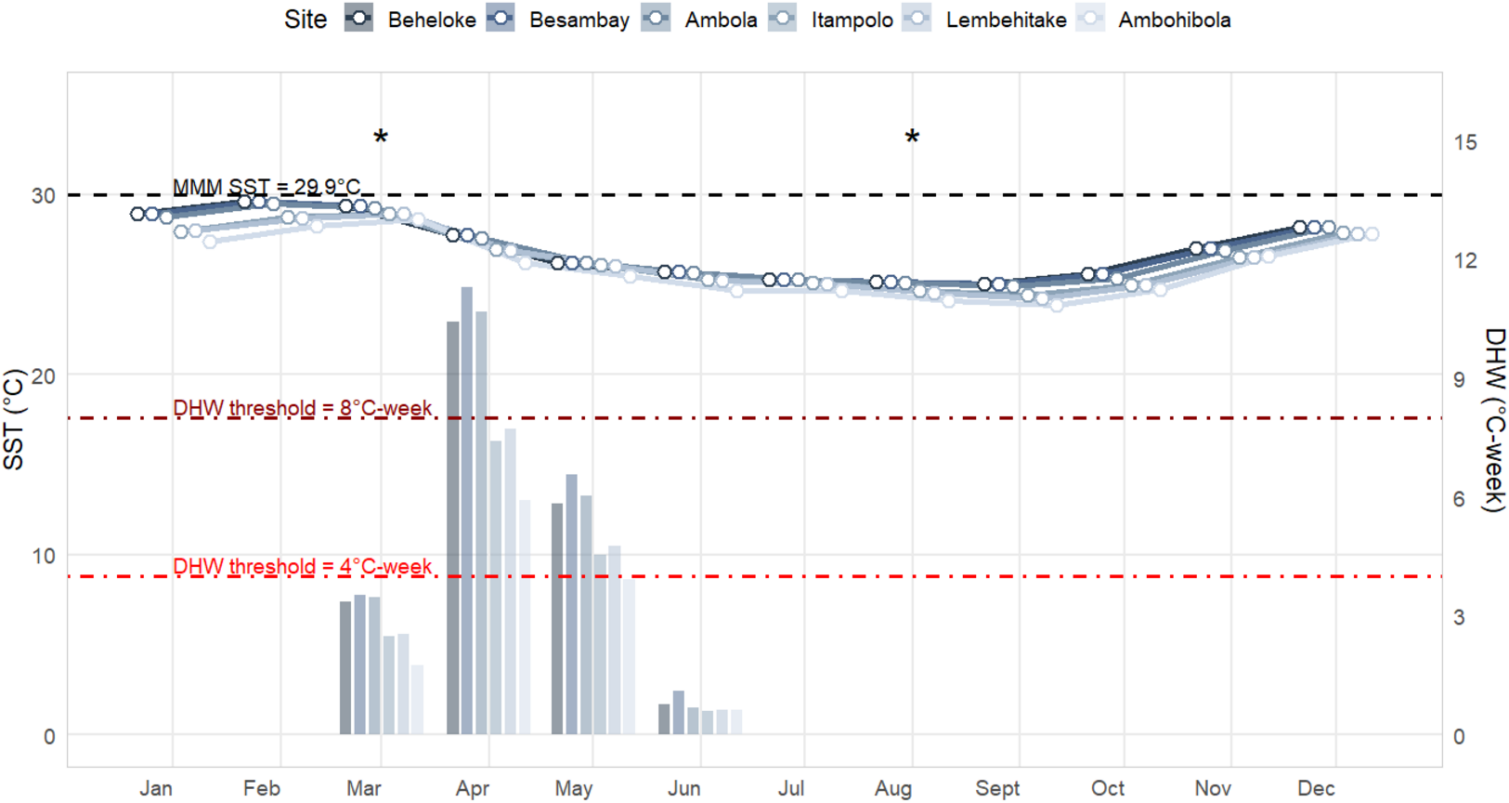
Sea surface temperature (SST; line plots) and degree heating weeks (DHW; bar plots) trends in 2024 across the six monitored reef sites. Sampling periods are marked with an asterisk (*).

Sea Surface Temperature (SST) and Degree Heating Week (DHW) patterns revealed a marked seasonal cycle across all monitored sites, with a clear north–south gradient (Fig. 3). The warmest conditions occurred between January and March, while the coolest months were between August and September. Maximum monthly mean (MMM) SSTs reached 29.6 °C at Beheloke and Ambola in February, remaining above 28.0 °C for at least three consecutive months (January–March). In contrast, cooler conditions were recorded at the three southern sites (Itampolo, Lembehitake, and Ambohibola), where MMM SSTs did not exceed 28.9 °C.

Such prolonged exposure to elevated temperatures likely contributed substantially to thermal stress accumulation. Indeed, DHW values indicated that thermal stress built up primarily between March and June, with the highest accumulation in April. In March, DHW peaked between 13.0 and 13.7 °C-weeks at Beheloke, Besambay, and Ambola, and between 9.7 and 9.8 °C-weeks at Itampolo and Lembehitake, while Ambohibola recorded the lowest value (6.8 °C-weeks). Accumulation persisted into April, with maximum DHW reaching 11.2 °C-weeks at Besambay and exceeding 10 °C-weeks at both Ambola and Beheloke. Moderate DHW levels (4–7 °C-weeks) remained through May at most sites before declining sharply in June (<1 °C-week) and remaining negligible from July, marking the end of the thermal stress season.

### Spatiotemporal patterns in coral bleaching response

**Figure 4:**
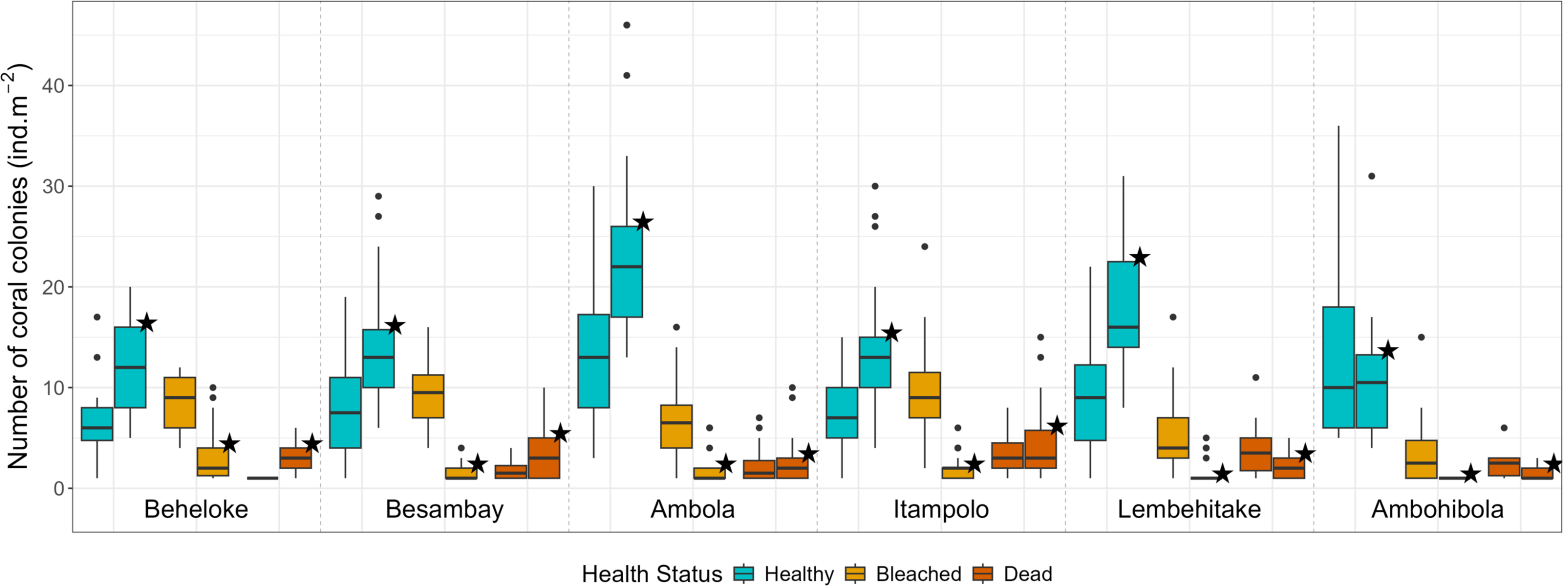
Coral colony abundance (all genera pooled) across reef sites, categorized by health status. Boxplots without stars represent data during the bleaching event, while boxplots marked with stars represent data after the event.

A total of 3,303 colonies (18.3 ± 0.4 colonies m^−2^ ± SE) were recorded during and 4,048 colonies (19.3 ±0.5 colonies m^−2^) after the bleaching event, while coral abundance among reef sites ranged from 24.0 ± 0.8 colonies m^−2^ in Ambola to 14.9 ± 1.4 colonies m^−2^ in Ambohibola (Fig. 4). A moderate spatiotemporal variation was detected when considering site (*χ*^2^ test, *p* = 0.021; Table 2) and sampling period (*χ*^2^ test, *p* = 0.013) as a single effect. However, *post-hoc* pairwise comparisons did not reveal significant differences among individual sites (Table S2), suggesting that the observed variability was mainly driven by overall spatial trends rather than pronounced site-specific contrasts.

In contrast, a stronger effect was attributed to the health status predictor, together with its interaction, in explaining the variability within our dataset (*χ*^2^ test, *p* < 0.001; Table 2). Almost all coral genera within the seascape exhibited higher bleaching response index (BRI) values during the bleaching event, followed by a consistent decrease afterward (Table 3). This trend was also reflected in site-specific bleaching prevalence and bleaching susceptibility index (BSI; Table 4). As shown by the evolution of these metrics, bleaching intensity was five-fold higher during the event, with 38.8% of corals exhibiting signs of bleaching in March, while only 6.0% remained bleached in August. A higher abundance of bleached coral colonies was then found during the event (EMMeans, *p* < 0.001; Fig. 4, Table S3), while healthy corals demonstrated a significantly higher abundance after the phenomenon (EMMeans, *p* < 0.001). However, the abundance of dead corals was not significantly different between the two periods (EMMeans, *p* = 0.167; Fig. 4, Table S3), despite an observed increase of 3.5% in dead colonies by the end of the thermal stress season.

**Table 2:** Type II Wald chi-square tests for the generalized linear mixed model (GLMM) on coral abundance (nbinom2 family).

**Predictors**	*χ* ^2^	**Df**	***P*-value**
Site	13.2	5	0.021^*^
Season	6.0	1	0.013^*^
Health status	1,100.7	2	<0.001^***^
Site x Health status	149.4	10	<0.001^***^
Season x Health status	380.1	2	<0.001^***^
Site x Season x Health status	70.2	15	<0.001^***^
Df. residuals	921		

**Notes.**

Model fit indices: AIC = 4,870.2, BIC = 5,055.1, logLik = −2,397.1, dispersion parameter = 9.34.

* *p* < 0.05.

** *p* < 0.01.

*** *p* < 0.001.

**Table 3:** Taxa-specific Bleaching Response Index (BRI) of scleractinian corals recorded during and after the event.

**Coral genera**	During		After		**Coral genera**	During		After	
	N	BRI	N	BRI		N	BRI	N	BRI
*Acropora*	829	38.3	864	24.0	*Millepora*	40	3.5	45	0.0
*Galaxea*	438	9.2	691	12.9	Branching *Porites*	39	25.6	45	4.8
*Pocillopora*	437	48.7	525	17.9	*Leptoseris*	26	17.6	51	6.6
*Stylophora*	177	24.8	292	27.3	*Acanthastrea*	37	37.2	34	27.0
*Favites*	209	11.6	253	7.8	*Goniastrea*	43	23.2	27	5.1
*Echinopora*	134	11.1	162	6.9	*Goniopora*	37	9.1	33	0.0
*Seriatopora*	80	64.0	206	24.0	*Platygyra*	29	12.4	41	6.3
Massive *Porites*	123	20.8	152	6.8	*Gardineroseris*	12	18.3	28	0.7
*Montipora*	132	7.2	142	7.0	*Fungia*	14	22.8	22	9.0
*Isopora*	70	12.5	104	1.3	*Turbinaria*	16	16.2	0	0.0
*Dipsastrea*	73	14.7	59	7.4	*Coscinaraea*	5	12.0	8	0.0
*Leptoria*	54	13.3	56	4.2	*Hydnophora*	0	0.0	12	8.3
*Pavona*	40	51.5	63	16.1	*Halomitra*	6	6.6	5	20.0
*Cyphastrea*	74	10.2	19	1.0	*Psammocora*	5	0.0	6	0.0
*Lobophyllia*	63	72.3	25	10.4	*Alveopora*	0	0.0	10	0.0
*Astreopora*	37	13.5	48	6.2	*Pleurogyra*	8	0.0	0	0.0

**Notes.**

N indicates the total count of coral colonies. Coral genera are ordered according to their total abundance (N).

**Table 4:** Summary of coral abundance, density, bleaching prevalence, Bleaching Response Index (BRI), and Bleaching Susceptibility Index (BSI) for each reef site.

Reef sites (Sampling period)	Coral colony abundance	Prevalence (%)	BRI	Coral density (colonies m^−2^ ± SE)	BSI
Beheloke (During)	358	58.1	16.7	14.9 ± 0.7	76.1
Beheloke (After)	593	30.0	21.7	17.4 ± 1.0	94.5
Besambay (During)	455	58.2	32.1	18.9 ± 1.0	160.6
Besambay (After)	659	20.9	17.5	17.3 ± 0.9	76.4
Ambola (During)	757	37.7	19.8	21.1 ± 1.0	73.3
Ambola (After)	1,033	13.7	11.3	26.7 ± 1.2	40.6
Itampolo (During)	759	62.5	56.3	19.6 ± 0.9	135.9
Itampolo (After)	717	27.7	42.0	18.3 ± 08	78.8
Lembehitake (During)	600	45.5	24.9	16.7 ± 0.9	125.6
Lembehitake (After)	788	11.2	9.2	20.4 ± 0.8	32.0
Ambohibola (During)	358	22.3	14.3	17.9 ± 2.3	84.1
Ambohibola (After)	238	9.6	9.8	11.9 ± 1.4	71.7

Across reef sites, Ambola and Ambohibola appeared the least impacted and most resistant to bleaching, with prevalence rates of only 22.3% and 37.7%, respectively, a BRI <20, and the lowest BSI scores recorded during the event (Fig. 4, Table 4). The entire dataset also revealed that Ambola supported a significantly higher abundance of healthy colonies compared to the five other reef sites (Table S4). Additionally, at Ambohibola, coral health status remained stable, with no significant differences detected between the two sampling periods, either for bleached colonies (EMMeans, *p* = 0.192; Table S5) or for healthy colonies (EMMeans, *p* = 0.061).

Conversely, Itampolo reefs were the most affected by coral bleaching, exhibiting the highest prevalence (62.5%) and the highest BRI (56.3; Table 4). This severe response was further associated with elevated coral mortality (19.5% of colonies recorded as dead after the event), with the genus *Lobophyllia* being particularly impacted (Table 3). Moreover, the abundance of dead corals was significantly higher at this site compared to more resilient ones, such as Ambola (EMMeans, *p* = 0.007) and Ambohibola (EMMeans, *p* = 0.005; Table S4). Besambay was the site where reefs were most susceptible to bleaching, exhibiting the highest BSI score of 160.6 (Table 4). Indeed, significantly more dead coral colonies were recorded at this site after the event compared to the previous sampling period (EMMeans, *p* = 0.006; Table S5). Itampolo and Lembehitake also show this high susceptibility, with BSI >100. Meanwhile, the BSI in Beheloke increased from 76.1 in March to 94.5 in August (Table 4).

Among the most abundant genera, *Pocillopora*, *Acropora*, and *Stylophora* recorded higher BRI scores of 48.7, 38.3, and 24.8, respectively. The BRI decreased markedly for the first two genera, while it increased from 24.8 to 27.3 after the event for *Stylophora*.

**Figure 5:**
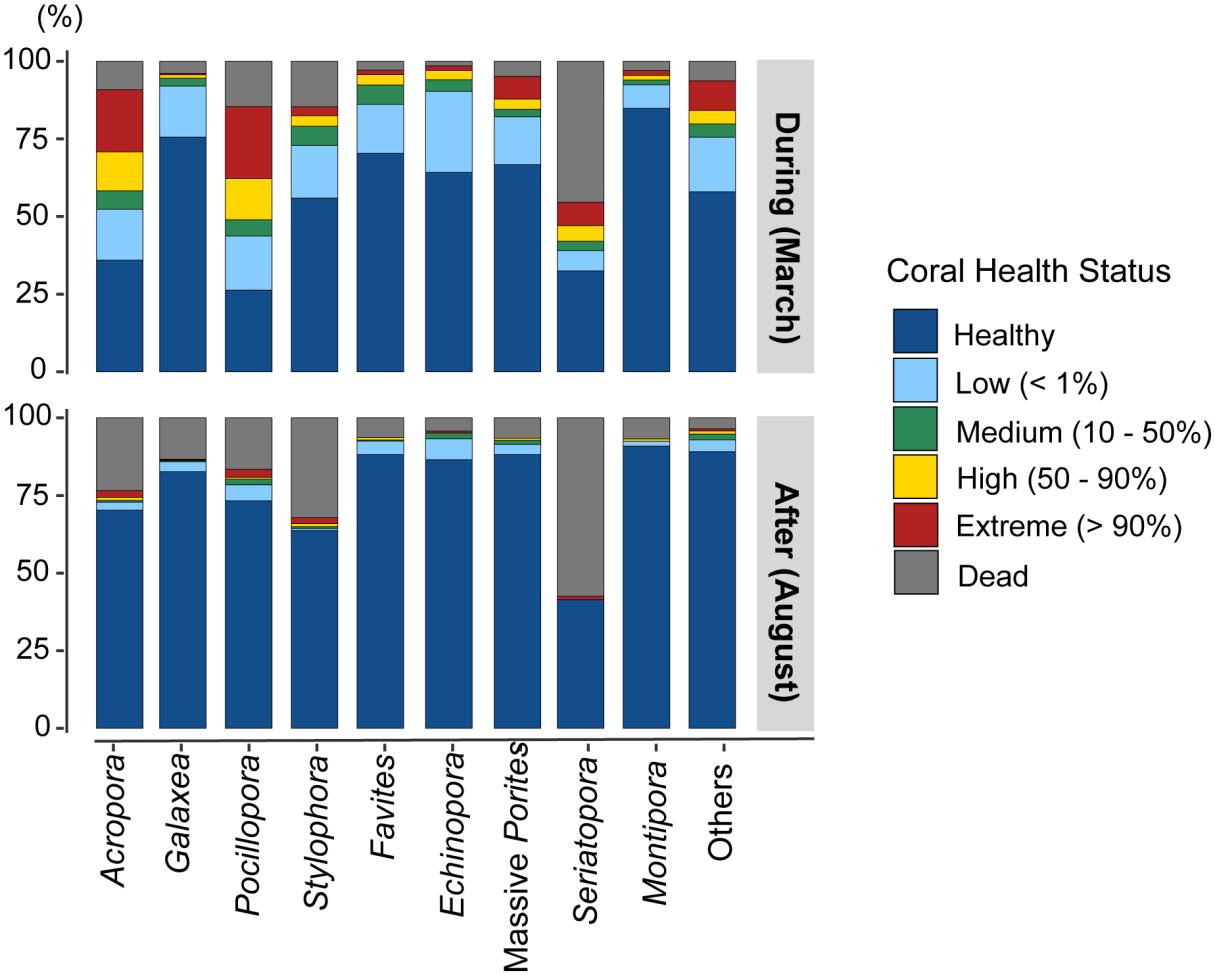
Proportion of bleaching observations by health status for the main coral genera and for less-represented genera grouped as “Others”, during and after the event.

Most coral genera exhibited a higher rate of bleaching during the event than after it (Fig. 5, Table 5), with the exceptions of *Galaxea* (*t*-test, *p* = 0.927), *Echinopora* (*p* = 0.262), and *Montipora* (*p* = 0.334). Furthermore, these three genera showed the lowest BRI (BRI < 20). There were more dead colonies in August for *Acropora* (*t*-test, *p* < 0.026) and *Stylophora* (*p* < 0.001). Although the genus *Seriatopora* demonstrated an important response during the event (BRI: 64.0), no differences were found between bleached colonies (*t*-test, *p* = 0.356) and dead colonies (*p* = 0.389) from March to August. Notably, the other less-represented genera showed a high bleaching rate in March (*t*-test, *p* < 0.001), followed by a significant mortality rate in August (*p* = 0.039), primarily linked with the high responses from genera such as *Lobophyllia* (highest BRI: 72.3) or *Pavona* (BRI: 51.5; Table 3).

## Discussion

Our findings underscore the complex dynamics of coral bleaching with patterns of both recovery and mortality varying across sites and coral taxa. We observed clear signs of bleaching severity during the event, followed by notable recovery in some areas and among certain genera.

### Thermal stress and coral bleaching response variability

Even though thermal stress, as here degree heating weeks (DHW), was not included in our model to explain coral bleaching variability, it remains a widely used metric for anticipating bleaching, with thresholds of 4.0 ^∘^C-weeks predicting severe bleaching and 8.0 ^∘^C-weeks predicting widespread mortality (Whitaker & DeCarlo, 2024). The higher the DHW, the greater the expected impact of bleaching (Heron et al., 2016). This is the case in our study, as among our six monitored sites, Besambay experienced the highest maximum DHW of 13.7 ^∘^C-weeks, where mortality was also the most significant following the bleaching event. Our general observations showed that 38.8% of corals in the Mahafaly seascape were bleached during our first survey (in March), despite DHW remaining below the 4.0 ^∘^C-week threshold. Severe bleaching has similarly been reported below this threshold in the Caribbean in 2005 (Eakin et al., 2010), in the Red Sea in 2016 (Monroe et al., 2018), and more recently at 1–2 °C-weeks on Kenyan reefs during the current 2024 mass bleaching event (Mayfield, 2025). It could be assumed that this percentage of bleached corals in the seascape might have been higher had our sampling been conducted one month later, when DHW reached <8.0 °C-weeks. Conversely, some reefs in the Pacific exhibit remarkable resilience despite DHW exceeding 15 ^∘^C-weeks in 2014 (Heron et al., 2016). Nevertheless, on Okinawa reefs, high DHW (13.2 ^∘^C-weeks) in 2022 led to 63% of corals bleaching and 14% experiencing bleaching-induced mortality (Afzal et al., 2024).

**Table 5:** Results of the Student’s *t*-test for matched samples on the relative abundance of Bleached (all levels) and Dead coral colonies during and after the event, for the nine main genera and “Others”.

Coral genera	Bleached coral colonies		Dead coral colonies	
	*t*	*p-*value	*t*	*p-*value
*Acropora*	−6.1	<0.001 ^***^	2.2	0.026 ^*^
*Galaxea*	0.9	0.927	0.9	0.338
*Pocillopora*	−4.3	<0.001 ^***^	7.8	0.073
*Stylophora*	−2.3	0.023 ^*^	3.8	**<0.001** ^***^
*Favites*	−3.3	0.001 ^**^	−0.8	0.451
*Echinopora*	−1.1	0.262	1.0	0.363
*Seriatopora*	−1.0	0.356	0.8	0.389
Massive *Porites*	−3.3	0.002 ^**^	−0.4	0.662
*Montipora*	−1.0	0.334	1.0	0.363
Others	−3.5	<0.001 ^***^	−2.1	0.039 ^*^

**Notes.**

Significant *P*-values (<0.05) are highlighted in bold (*: <0.05, **: <0.01, ***: <0.001).

Our observations in the Mahafaly seascape reefs align with these global nuances. While DHW broadly predicted bleaching onset, the severity and recovery trajectories varied markedly among sites. Some reefs, such as Ambola (maximum DHW 13.4 ^∘^C-weeks), exhibited strong resilience despite high heat exposure. In contrast, other reefs, such as Itampolo (maximum DHW 9.6 ^∘^C-weeks), were disproportionately affected even under moderate DHW. This site-specific variability underscores that, although DHW is a robust indicator of thermal stress, local environmental conditions and reef-specific characteristics ultimately determine ecological outcomes.

### Environmental drivers and species-specific bleaching responses

As evidenced by several studies, bleaching response at the assemblage level can vary across multiple scales (Hughes et al., 2018a; Szereday, Voolstra & Amri, 2024; Yasunaka, Kurihara & Doi, 2025). These variations are mostly linked to the taxonomic composition of reef sites. In the SWIO region, previous research shows that bleaching responses have been attributed to the initial species composition of coral communities, with sites dominated by susceptible species (*e.g., Acropora and Pocillopora*) being more likely to experience bleaching (McClanahan et al., 2007a; Gudka et al., 2020). This pattern is clearly corroborated by our results. For example, Itampolo, one of the most affected reefs, is composed of 20.2% *Acropora*, 13.4% *Pocillopora*, and 14.4% *Stylophora*. Furthermore, Besambay, the most susceptible reef, consists of 30.2% *Acropora*. Interestingly, reefs in Ambohibola have the highest relative abundance of *Acropora* (31.0%). Although this is the only reef site located in a shallow habitat, it is the least affected by bleaching. With the lowest thermal stress recorded at this southernmost site, this exception can be explained by high hydrodynamics and turbidity of the seawater in the area, with sediments primarily carried by the Linta River. The protective effect of turbid habitats on bleaching impacts has also been observed in several reefs worldwide across different bleaching events (Marshall & Baird, 2000; Morgan et al., 2017; Teixeira et al., 2019).

Several studies have also shown that coral assemblages that have persisted in frequently disturbed environments are more resilient (Barkley et al., 2018; Graham et al., 2015; Obura, Bigot & Benzoni, 2018; Page et al., 2021). On one hand, some corals are capable of acclimatization and adaptation, potentially giving rise to more resilient generations (Pratchett et al., 2013; Torda et al., 2017; Fuller et al., 2020). On the other hand, the composition of coral assemblages may shift from being dominated by competitive and sensitive taxa (*e.g.*, branching corals), to assemblages dominated by stress-tolerant groups (*e.g*., crustose and massive corals; Bento et al., 2016; Hughes et al., 2018b; Romero-Torres et al., 2020). Unfortunately, limited research has been conducted on the coral reefs in the south of Toliara, within the Mahafaly seascape, to understand the evolutionary changes potentially induced by coral bleaching and to link the reef sites responses to the ongoing event.

### Potential role of the Mahafaly seascape as climate refugia

Despite the high response of thermally sensitive taxa and some surveyed reef sites, an insignificant coral mortality rate was observed throughout the Mahafaly seascape after the bleaching event. This highlights the potential for coral reefs in this region to rebound, when bleaching intensity is not overwhelming. The severity of bleaching and subsequent mortality (38.8% of corals experienced bleaching and 3.5% mortality, likely attributable to the event) appears relatively low compared to other reef sites and past bleaching events, both nationally and across other Indo-Pacific reefs. For instance, during the first mass bleaching event, approximately 30.0% of coral cover was bleached, with 14.0% mortality recorded in some reefs along the western coast of Madagascar (near the Belo sur Mer region; Ahamada et al., 2002). During the second event, the SWIO region experienced significant impacts, with 17.5% coral mortality reported in Mayotte (Obura, Bigot & Benzoni, 2018). The third event was one of the most severe, with 40.0% of reefs in Madagascar showing signs of bleaching (Gudka et al., 2018). At the regional scale, 64.0% of SWIO reef sites experienced bleaching, and 9.0% suffered coral mortality (Gudka et al., 2020). Similarly, in other Indo-Pacific reefs, nearly half of the corals bleached, with associated mortality estimated at approximately 9.8% (Rodgers et al., 2017).

The opportunity for reefs in the Mahafaly seascape lies in their geographical location, which places them near subtropical reefs, where SST and thermal stress are generally lower (Ho et al., 2024). Low-latitude coral regions are particularly vulnerable to thermal stress and are projected to experience severe-bleaching conditions for over three months per year, with early onsets likely as soon as 2035 (Mellin et al., 2024). The increases in severity and duration of thermal stress in these reefs can subsequently affect coral life cycle events, particularly if bleaching coincides with critical periods like coral spawning or larval recruitment (Hughes et al., 2019). In this context, reefs in this seascape could serve as climate refugia, playing an important role in the future for the persistence of coral reefs and deserving particular attention in terms of conservation efforts (McClanahan et al., 2023; Dimitrijević, Santodomingo & Kiessling, 2024). An additional and important factor for the reefs in this seascape is their distance from the city of Toliara, which reduces anthropogenic influences from tourism and fishing, as well as sedimentation impacts, compared to closer reefs. Gough et al. (2009) suggest that this distance is associated with an increase in reef species richness and improved reef health.

### Implication for effective coral reef management

The contrasting patterns of bleaching observed in reef sites within the Mahafaly seascape provide a clearer understanding to refine management measures and implement targeted actions on reefs within LMMAs. We can infer that the strong response of Itampolo and Beheloke reefs is due to the high human population numbers (see Table 1), which may increase pressures on these reefs. Another additional hypothesis for Itampolo is the low larval supply to this reef due to its location, which is relatively protected from currents. This could reduce coral recruitment, one of the key pillars of reef resilience (Botosoamananto et al., 2024). Decisions need to be taken by managers and local community committees involved in LMMAs regarding all critical locations, in particular by regulating fishing on key functional groups, such as herbivorous fish. These organisms often mediate coral-algal interactions in favor of reef-building corals (Cook et al., 2024). This approach has already shown evidence of its effectiveness in some reefs in the Pacific following the 2016 bleaching event (Chung et al., 2019; Donovan et al., 2023). There is also a need to develop holistic coral conservation strategies. This includes the establishment and enforcement of regulations within existing local community-managed permanent reserves (*e.g.,* Ranolaly and Kirinjo reserves in Beheloke and Besambay, respectively). Active reef restoration efforts, such as artificial reefs and coral transplantation programs, should be prioritized, especially for reefs like those in Itampolo, where coral recruitment may be limited due to local environmental conditions. Such approaches can enhance reef habitat complexity and accelerate recovery (Boström-Einarsson et al., 2020), but they are often costly, labor-intensive, and show variable long-term success, particularly under ongoing climate stress (Mulà et al., 2025). Furthermore, coral taxa responses to thermal stress can serve as a guideline for selecting species that are more resistant and suitable for transplantation. Reef conservation efforts in this region should not only focus on regulating marine activities but also address land-based threats, such as deforestation, agricultural runoff, coastal development, and human population dynamics.

### Study limitations and research perspectives

While our findings offer insights into site- and taxa-specific variability in bleaching responses, limitations such as fine-scale spatial constraints remain. Our sampling was restricted to reef slope habitats and a single depth stratum, which may limit the ecological representativeness of our results within the seascape. This underscores the need for further testing across a broader range of reef habitats (*e.g*., inner reef, back reef, reef crest) and depth zones. Another limitation is that this study was not designed to evaluate the influence of existing management interventions, such as comparing reef conditions inside and outside managed areas, despite the relevance of such assessments. While direct mitigation of bleaching through protection alone is debated, MPAs are widely recognized for supporting reef recovery processes (Graham et al., 2008; Mumby et al., 2022). Consistent with this, national-level evidence from northwest Madagascar (Randrianarivo et al., 2024) suggests that well-designed and effectively enforced management can enhance coral community resilience by reducing local anthropogenic stressors.

## Conclusions

Patterns of coral bleaching in the Mahafaly seascape reveal both resilience and vulnerability within coral assemblages. The variation in bleaching severity and recovery across different reef sites emphasizes the significant role of thermal stress, species composition and local environmental factors. Reefs experiencing higher degree heating weeks and hosting abundant thermally sensitive taxa showed greater susceptibility to bleaching, while other sites with relatively protective factors, such as turbidity and high hydrodynamics, demonstrated better resilience. Interestingly, coral mortality rates across the seascape remained relatively low, indicating that coral reefs in the region have a high potential for recovery. With its geographical advantages and existing management systems (Biosphere Reserve, MPAs and LMMAs), the Mahafaly seascape reefs could serve as an important refugium for coral reefs in the future. By giving these valuable insights into the responses of coral assemblages in this seascape during a critical bleaching event, we hope to inform future research and conservation efforts aimed at enhancing the resilience of coral reefs in a rapidly changing world.

## Supplemental Information

10.7717/peerj.20319/supp-1 Supplemental Information 1 DHARMa Residual Diagnostics for generalized linear mixte model fit QQ plot of residuals with non-significant results from the Kolmogorov-Smirnov, dispersion, and outlier tests, indicating no major deviations.

10.7717/peerj.20319/supp-2 Supplemental Information 2 Multicollinearity diagnostics for model predictors: VIF, adjusted VIF, and tolerance

10.7717/peerj.20319/supp-3 Supplemental Information 3 Pairwise site contrasts from the generalized linear mixed model on coral abundance Positive estimates indicate higher values in the first site compared to the second.

10.7717/peerj.20319/supp-4 Supplemental Information 4 Pairwise contrasts of overal coral health status between sampling periods Positive estimates indicate higher values in the first site compared to the second. Significant *p* -values (<0.05) are highlighted in bold (*: <0.05, **: <0.01, ***: <0.001).

10.7717/peerj.20319/supp-5 Supplemental Information 5 Pairwise comparisons of reef sites for each coral health category (Bleached, Dead, Healthy), derived from the generalized linear mixed model Positive estimates indicate higher values in the first site compared to the second. Significant *p*-values (<0.05) are highlighted in bold (*: <0.05, **: <0.01, ***: <0.001).

10.7717/peerj.20319/supp-6 Supplemental Information 6 Pairwise contrasts of coral health status between sampling periods for each site Positive estimates indicate higher values in the first site compared to the second.Significant*p*-values (<0.05) are highlighted in bold (*: <0.05, **: <0.01, ***: <0.001).

10.7717/peerj.20319/supp-7 Supplemental Information 7 Bleaching MHF 2024 The abundance and bleaching status of different coral genera observed across various sites, sampling stations, seasons, and quadrants.

## Additional Information and Declarations

### Data Availability

The following information was supplied regarding data availability: The raw data is available in the Supplemental Files.
