# Supplementary figures and images for: Coral reefs in the Mahafaly Seascape (SW Madagascar) as potential climate refugia following the 2024 mass bleaching event
Source: PeerJ. 2025 Nov 25;13:e20319. doi: 10.7717/peerj.20319 (PMC12662060; doi:10.7717/peerj.20319)

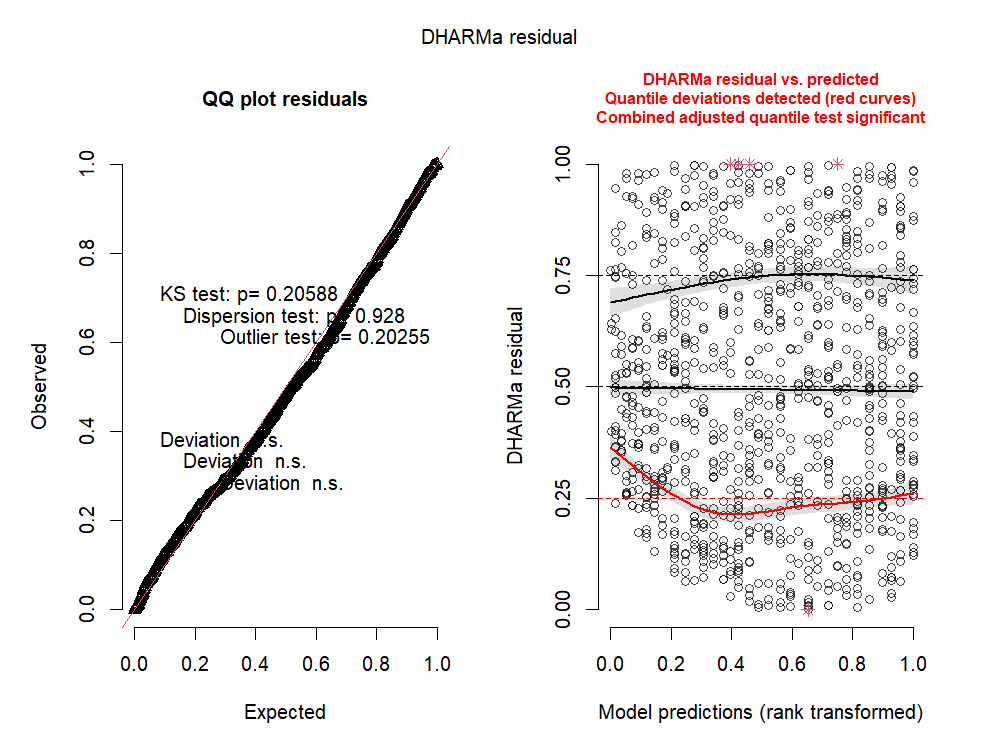

Supplement: Supplemental Information 1 — QQ plot of residuals with non-significant results from the Kolmogorov-Smirnov, dispersion, and outlier tests, indicating no major deviations. [file peerj-13-20319-s001.png]
