# Supplementary material for: Coral reefs in the Mahafaly Seascape (SW Madagascar) as potential climate refugia following the 2024 mass bleaching event
Source: PeerJ. 2025 Nov 25;13:e20319. doi: 10.7717/peerj.20319 (PMC12662060; doi:10.7717/peerj.20319)
Supplement: Supplemental Information 2 [file peerj-13-20319-s002.docx]

| **Term** | **VIF** | **VIF 95% CI** | **Adjusted VIF** | **Tolerance** | **Tolerance 95% CI** |
| --- | --- | --- | --- | --- | --- |
| Health status | 1.16 | [1.10, 1.27] | 1.08 | 0.86 | [0.79, 0.91] |
| Season | 1.15 | [1.09, 1.26] | 1.07 | 0.87 | [0.80, 0.92] |
| Site | 1.04 | [1.01, 1.21] | 1.02 | 0.96 | [0.83, 0.99] |
| DHW | — | — | — | — | — |
