# Supplementary material for: Coral reefs in the Mahafaly Seascape (SW Madagascar) as potential climate refugia following the 2024 mass bleaching event
Source: PeerJ. 2025 Nov 25;13:e20319. doi: 10.7717/peerj.20319 (PMC12662060; doi:10.7717/peerj.20319)
Supplement: Supplemental Information 3 — Positive estimates indicate higher values in the first site compared to the second. [file peerj-13-20319-s003.docx]

| **Site contrast** | **Estimate** | **SE** | **df** | **z.ratio** | ***p*-value** |
| --- | --- | --- | --- | --- | --- |
| Ambohibola – Ambola | -0.38 | 0.21 | Inf | -1.81 | 0.069. |
| Ambohibola – Beheloke | -0.15 | 0.23 | Inf | -0.66 | 0.503 |
| Ambohibola – Besambay | -0.21 | 0.21 | Inf | -1.01 | 0.310 |
| Ambohibola – Itampolo | -0.39 | 0.21 | Inf | -1.86 | 0.062. |
| Ambohibola – Lembehitake | -0.26 | 0.21 | Inf | -1.25 | 0.208 |
| Ambola – Beheloke | 0.22 | 0.13 | Inf | 1.66 | 0.095 |
| Ambola – Besambay | 0.16 | 0.09 | Inf | 1.70 | 0.088 |
| Ambola – Itampolo | -0.007 | 0.09 | Inf | -0.07 | 0.94 |
| Ambola – Lembehitake | 0.11 | 0.09 | Inf | 1.19 | 0.232 |
| Beheloke – Besambay | -0.06 | 0.13 | Inf | -0.43 | 0.664 |
| Beheloke – Itampolo | -0.23 | 0.13 | Inf | -1.75 | 0.080 |
| Beheloke – Lembehitake | -0.11 | 0.13 | Inf | -0.81 | 0.417 |
| Besambay – Itampolo | -0.17 | 0.09 | Inf | -1.84 | 0.065 |
| Besambay – Lembehitake | -0.05 | 0.1 | Inf | -0.51 | 0.606 |
| Itampolo – Lembehitake | 0.12 | 0.09 | Inf | 1.31 | 0.188 |
