# Supplementary material for: Coral reefs in the Mahafaly Seascape (SW Madagascar) as potential climate refugia following the 2024 mass bleaching event
Source: PeerJ. 2025 Nov 25;13:e20319. doi: 10.7717/peerj.20319 (PMC12662060; doi:10.7717/peerj.20319)
Supplement: Supplemental Information 4 — Positive estimates indicate higher values in the first site compared to the second. Significant p -values (<0.05) are highlighted in bold (*: <0.05, **: <0.01, ***: <0.001). [file peerj-13-20319-s004.docx]

| **Health status contrast between sampling period** | **Estimate** | **SE** | **df** | **z.ratio** | **p.value** |
| --- | --- | --- | --- | --- | --- |
| \| Bleached (After - During)  \| \| --- \| | -1.34 | 0.19 | Inf | -7.03 | **<0.001 ***** |
| Dead (After - During) | 0.18 | 0.17 | Inf | 1.37 | 0.167 |
| Healthy (After – During) | 0.46 | 0.04 | Inf | 9.86 | **<0.001***** |
