# Supplementary material for: Coral reefs in the Mahafaly Seascape (SW Madagascar) as potential climate refugia following the 2024 mass bleaching event
Source: PeerJ. 2025 Nov 25;13:e20319. doi: 10.7717/peerj.20319 (PMC12662060; doi:10.7717/peerj.20319)
Supplement: Supplemental Information 5 — Positive estimates indicate higher values in the first site compared to the second. Significant p-values (<0.05) are highlighted in bold (*: <0.05, **: <0.01, ***: <0.001). [file peerj-13-20319-s005.docx]

| **Contrast** | **Estimate** | **SE** | **df** | **z.ratio** | **p-value** |
| --- | --- | --- | --- | --- | --- |
| **Bleached** | | | | | |
| Ambohibola – Ambola | -0.58 | 0.54 | Inf | -1.07 | 0.281 |
| Ambohibola – Beheloke | -0.96 | 0.54 | Inf | -1.78 | 0.074 |
| Ambohibola – Besambay | -0.62 | 0.54 | Inf | -1.14 | 0.252 |
| Ambohibola – Itampolo | -0.78 | 0.54 | Inf | -1.44 | 0.148 |
| Ambohibola – Lembehitake | -0.38 | 0.54 | Inf | -0.70 | 0.478 |
| Ambola – Beheloke | -0.37 | 0.14 | Inf | -2.64 | **0.008 **** |
| Ambola – Besambay | -0.03 | 0.15 | Inf | -0.23 | 0.815 |
| Ambola – Itampolo | -0.19 | 0.14 | Inf | -1.36 | 0.172 |
| Ambola – Lembehitake | 0.20 | 0.16 | Inf | 1.22 | 0.220 |
| Beheloke – Besambay | 0.34 | 0.14 | Inf | 2.33 | **0.019*** |
| Beheloke – Itampolo | 0.18 | 0.13 | Inf | 1.38 | 0.165 |
| Beheloke – Lembehitake | 0.58 | 0.15 | Inf | 3.80 | **<0.001 ***** |
| Besambay – Itampolo | -0.16 | 0.14 | Inf | -1.08 | 0.277 |
| Besambay – Lembehitake | 0.23 | 0.16 | Inf | 1.42 | 0.153 |
| Itampolo – Lembehitake | 0.39 | 0.15 | Inf | 2.59 | **0.009 **** |
| **Dead** | | | | | |
| Ambohibola – Ambola | -0.21 | 0.22 | Inf | -0.96 | 0.335 |
| Ambohibola – Beheloke | 0.16 | 0.36 | Inf | 0.44 | 0.660 |
| Ambohibola – Besambay | -0.18 | 0.22 | Inf | -0.79 | 0.426 |
| Ambohibola – Itampolo | -0.59 | 0.21 | Inf | -2.76 | **0.005 **** |
| Ambohibola – Lembehitake | -0.36 | 0.22 | Inf | -1.63 | 0.102 |
| Ambola – Beheloke | 0.37 | 0.33 | Inf | 1.13 | 0.256 |
| Ambola – Besambay | 0.03 | 0.15 | Inf | 0.21 | 0.832 |
| Ambola – Itampolo | -0.37 | 0.14 | Inf | -2.69 | **0.007 **** |
| Ambola – Lembehitake | -0.14 | 0.15 | Inf | -0.97 | 0.329 |
| Beheloke – Besambay | -0.34 | 0.33 | Inf | -1.02 | 0.305 |
| Beheloke – Itampolo | -0.75 | 0.32 | Inf | -2.31 | **0.020 *** |
| Beheloke – Lembehitake | -0.52 | 0.33 | Inf | -1.58 | 0.113 |
| Besambay – Itampolo | -0.41 | 0.14 | Inf | -2.79 | **0.005 **** |
| Besambay – Lembehitake | -0.18 | 0.15 | Inf | -1.14 | 0.250 |
| Itampolo – Lembehitake | 0.23 | 0.13 | Inf | 1.67 | 0.093 |
| **Healthy** | | | | | |
| Ambohibola – Ambola | -0.34 | 0.11 | Inf | -2.96 | **0.003 **** |
| Ambohibola – Beheloke | 0.34 | 0.12 | Inf | 2.71 | **0.006 **** |
| Ambohibola – Besambay | 0.15 | 0.12 | Inf | 1.30 | 0.193 |
| Ambohibola – Itampolo | 0.20 | 0.11 | Inf | 1.73 | 0.082 |
| Ambohibola – Lembehitake | -0.05 | 0.11 | Inf | -0.43 | 0.661 |
| Ambola – Beheloke | 0.68 | 0.10 | Inf | 6.57 | **<0.001 ***** |
| Ambola – Besambay | 0.50 | 0.10 | Inf | 4.97 | **<0.001 ***** |
| Ambola – Itampolo | 0.55 | 0.097 | Inf | 5.69 | **<0.001 ***** |
| Ambola – Lembehitake | 0.29 | 0.096 | Inf | 3.06 | **0.002 **** |
| Beheloke – Besambay | -0.18 | 0.11 | Inf | -1.62 | 0.104 |
| Beheloke – Itampolo | -0.13 | 0.10 | Inf | -1.24 | 0.212 |
| Beheloke – Lembehitake | -0.39 | 0.10 | Inf | -3.70 | **0.002 **** |
| Besambay – Itampolo | 0.04 | 0.10 | Inf | 0.45 | 0.650 |
| Besambay – Lembehitake | -0.21 | 0.10 | Inf | -2.05 | **0.040 *** |
| Itampolo – Lembehitake | -0.25 | 0.098 | Inf | -2.6 | **0.008 **** |
