# Supplementary material for: Coral reefs in the Mahafaly Seascape (SW Madagascar) as potential climate refugia following the 2024 mass bleaching event
Source: PeerJ. 2025 Nov 25;13:e20319. doi: 10.7717/peerj.20319 (PMC12662060; doi:10.7717/peerj.20319)
Supplement: Supplemental Information 6 — Positive estimates indicate higher values in the first site compared to the second.Significantp-values (<0.05) are highlighted in bold (*: <0.05, **: <0.01, ***: <0.001). [file peerj-13-20319-s006.docx]

| **Health status contrast between sampling period across site** | **Estimate** | **SE** | **df** | **z.ratio** | ***p-*value** |
| --- | --- | --- | --- | --- | --- |
| Bleached corals in Beheloke  (After - During) | -0.96 | 0.15 | Inf | -6.08 | **<0.001 ***** |
| Bleached corals in Besambay  (After - During) | -1.85 | 0.20 | Inf | -8.93 | **<0.001 ***** |
| Bleached corals in Ambola  (After - During) | -1.19 | 0.19 | Inf | -6.01 | **<0.001 ***** |
| Bleached corals in Itampolo  (After - During) | -1.56 | 0.16 | Inf | -9.64 | **<0.001 ***** |
| Bleached corals in Lembehitake  (After - During) | -1.10 | 0.22 | Inf | -4.96 | **<0.001 ***** |
| Bleached corals in Ambohibola  (After – During) | -1.38 | 1.06 | Inf | -1.30 | 0.192 |
| Dead corals in Beheloke  (After - During) | 1.07 | 0.61 | Inf | 1.73 | 0.082 |
| Dead corals in Besambay  (After - During) | 0.57 | 0.21 | Inf | 2.70 | **0.006**** |
| Dead corals in Ambola  (After - During) | 0.20 | 0.19 | Inf | 1.06 | 0.285 |
| Dead corals in Itampolo  (After - During) | 0.20 | 0.15 | Inf | 1.34 | 0.059. |
| Dead corals in Lembehitake  (After - During) | -0.41 | 0.18 | Inf | 2.20 | **0.027*** |
| Dead corals in Ambohibola  (After - During) | -0.52 | 0.36 | Inf | -1.44 | 0.147 |
| Healthy corals in Beheloke  (After - During) | 0.66 | 0.12 | Inf | 5.11 | **<0.001 ***** |
| Healthy corals in Besambay  (After - During) | 0.54 | 0.12 | Inf | 4.54 | **<0.001 ***** |
| Healthy corals in Ambola  (After - During) | 0.57 | 0.09 | Inf | 6.02 | **<0.001 ***** |
| Healthy corals in Itampolo  (After - During) | 0.58 | 0.10 | Inf | 5.59 | **<0.001 ***** |
| Healthy corals in Lembehitake  (After - During) | 0.68 | 0.10 | Inf | 6.82 | **<0.001 ***** |
| Healthy corals in Ambohibola  (After - During) | -0.25 | 0.13 | Inf | -1.86 | 0.061 |
